# The Physical Activity Tracker Testing in Youth (P.A.T.T.Y.) Study: Content Analysis and Children’s Perceptions

**DOI:** 10.2196/mhealth.6347

**Published:** 2017-04-28

**Authors:** Brittany Masteller, John Sirard, Patty Freedson

**Affiliations:** ^1^ Department of Kinesiology University of Massachusetts Amherst Amherst, MA United States

**Keywords:** child, physical activity, qualitative research

## Abstract

**Background:**

Activity trackers are widely used by adults and several models are now marketed for children.

**Objective:**

The aims of this study were to (1) perform a content analysis of behavioral change techniques (BCTs) used by three commercially available youth-oriented activity trackers and (2) obtain feedback describing children’s perception of these devices and the associated websites.

**Methods:**

A content analysis recorded the presence of 36 possible BCTs for the MovBand (MB), Sqord (SQ), and Zamzee (ZZ) activity trackers. In addition, 16 participants (mean age 8.6 years [SD 1.6]; 50% female [8/16]) received all three trackers and were oriented to the devices and websites. Participants were instructed to wear the trackers on 4 consecutive days and spend ≥10 min/day on each website. A cognitive interview and survey were administered when the participant returned the devices. Qualitative data analysis was used to analyze the content of the cognitive interviews. Chi-square analyses were used to determine differences in behavioral monitoring and social interaction features between websites.

**Results:**

The MB, SQ, and ZZ devices or websites included 8, 15, and 14 of the possible 36 BCTs, respectively. All of the websites had a behavioral monitoring feature (charts for tracking activity), but the percentage of participants indicating that they “liked” those features varied by website (MB: 8/16, 50%; SQ: 6/16, 38%; ZZ: 11/16, 69%). Two websites (SQ and ZZ) included an “avatar” that the user could create to represent themselves on the website. Participants reported that they “liked” creating and changing their avatar (SQ: 12/16, 75%, ZZ: 15/16, 94%), which was supported by the qualitative analyses of the cognitive interviews. Most participants (75%) indicated that they would want to wear the devices more if their friends were wearing a tracker. No significant differences were observed between SQ and ZZ devices in regards to liking or use of social support interaction features (P=.21 to .37).

**Conclusions:**

The websites contained several BCTs consistent with previously identified strategies. Children “liked” the social aspects of the websites more than the activity tracking features. Developers of commercial activity trackers for youth may benefit from considering a theoretical perspective during the website design process.

## Introduction

### Background

The importance of engaging in daily physical activity (PA) to attenuate the prevalence of preventable chronic disease is well documented. To that end, it is recommended that children and adolescents engage in at least 60 min of moderate-to-vigorous intensity PA every day to maintain or improve health status [1]. However, only 42% of children aged 6-11 years meet this recommendation [2]. To offset the rise of unhealthy trends in youth, new strategies are continually being proposed to modify these unhealthy behaviors.

One of the new approaches being explored is to have youth use trackers to self-manage activity behavior. Previous studies have been successful using pedometers as a tool to promote behavior change [3,4]. Electronic self-monitoring devices, such as fitness trackers, have become very popular with recent reports stating that sales have been more than doubled since 2014 [5]. These devices are targeted at various populations, including children. Consumer activity trackers contain accelerometers and provide measures of activity level to the user with the intent to increase PA behavior. Some studies in adults have shown increases in activity when using a device such as a pedometer or activity tracker [6-8]. However, the capability of these devices to improve behavior relies partially on the device’s ability to engage users to encourage and support positive behavior change [9,10].

Using theory to guide interventions designed to change health behaviors in adults and youth is well-supported [11-14]. Theoretical models provide mediating variables that can be assessed to better understand intervention success or failure and allow the research to be replicated or modified in future implementations. To what extent these constructs are incorporated into activity tracker websites remains unclear. In particular, there is a lack of research surrounding these devices when used with children.

With the potential for consumer activity trackers to increase PA, it is important to evaluate the behavioral change techniques (BCTs) that are incorporated into these devices to identify how changes (if any) are produced and how it can be improved. BCTs can be defined as observable and replicable components of behavior change interventions [14]. BCTs can be implemented alone or combined to elicit a specific behavior change in intervention areas such as PA.

Middelweerd et al [15] recently performed a content analysis on mobile apps to promote PA in adults. However, limited research has evaluated the presence of BCTs incorporated into activity trackers, particularly in children. Of the PA tracking devices that are currently marketed to children, only the Zamzee (ZZ) has been evaluated, although the device maker conducted this study [16]. The results of this study suggested that users of the ZZ (54-68%) participated in more moderate to vigorous PA than their control group counterparts (*P*<.001). Additional independent research is needed to inform the community about how well this device and others incorporate BCTs to stimulate behavior change in this population.

### Aims of This Study

The aims of this exploratory study were to (1) perform a content analysis to evaluate the BCTs present in three commercially available youth-oriented activity trackers and (2) to obtain feedback and information describing the children’s perception of these devices and the associated websites.

## Methods

### Participants

Children (N=16; age 8.6 years [SD 1.6]; 50% female) were recruited through posted flyers and word of mouth. Inclusion criteria included age (6-11 years) and no physical or mental disabilities that would interfere with the child’s ability to perform PA, understand website navigation, or follow protocol instructions. Children were recruited to reflect the elementary school age range, which appears to be a target demographic for the activity trackers tested in this study. The Institutional Review Board approved this study. Written informed consent was obtained from parents, and assent forms were read to each child before any data collection.

### Procedures

Before recruiting participants, a content analysis was completed for each tracker and associated website to identify theory-based BCTs. Following the content analysis, children were recruited to participate in the free-living portion of the study. At an initial visit, each participant was asked to simultaneously wear three commercially available PA trackers on four consecutive days. Children were instructed on use of the trackers, given a brief introduction to each website’s features, and provided with instructions on uploading activity tracker data to the websites. Participants were instructed to spend a minimum of 10 min each day on each of the three websites. Parents were provided a log to record the amount of time that the child had spent on each website. After 4 days of wear, children returned all devices and then completed cognitive interviews. Children then completed a survey based on awareness and perception of specific BCTs for each website.

### Physical Activity Trackers

**Figure 1:**
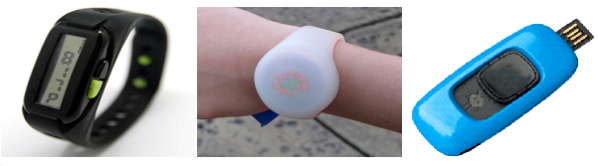
From left to right, the MovBand (MB), Sqord (SQ), and Zamzee (ZZ) activity trackers.

This investigation focused on three commercially available devices (Figure 1). The Sqord (SQ: Sqord, Inc, Durham, NC, USA) is a wrist-worn device similar to a watch that lacks a display. The MovBand (MB: MovBand, LLC, Brecksville, OH, USA) is another wrist-worn device that displays the time and “Moves” or “Steps.” Finally, the ZZ (a project of HopeLab, Redwood City, CA, USA) is a hip-worn device that uses a built-in clip to attach to a user’s waistband, pants pocket, or other location. For this study, the ZZ was clipped to an elastic strap with the device positioned over the child’s hip.

**Figure 2:**
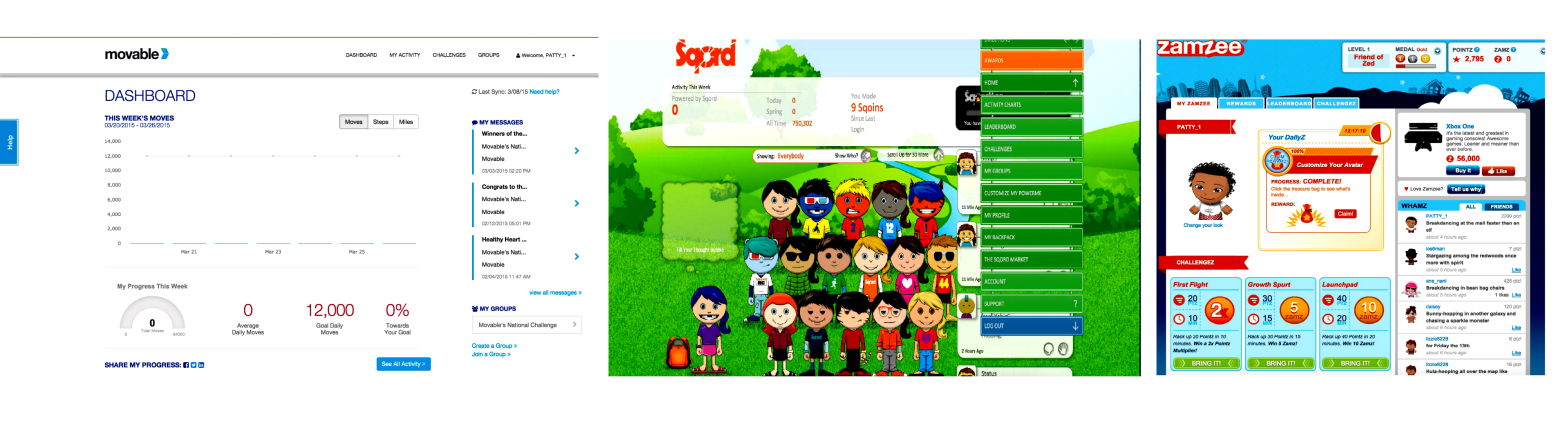
The home pages of the MovBand (MB), Sqord (SQ), and Zamzee (ZZ) websites.

Each device used specific terms or language in the corresponding website. Images of the home screen for each device can be seen in Figure 2. Each device tallied points throughout the day and upon syncing the device, points that were accumulated since the last upload would appear for the user to view. For the SQ, the metrics were called “activity points” and “sqoins.” If you earned enough “sqoins,” you could purchase items to put in your “backpack” (eg, clothing for website avatar). For the ZZ, the metric was called “pointz,” and if you accumulated enough “pointz,” you could then earn the more valuable metric of “zamz.” Similar to the SQ, the ZZ website had a feature where you could purchase “rewards” when enough zamz were earned. Once the users totaled the minimum number of zamz, they could purchase virtual and actual items (eg, clothing for website avatar, shoelaces); actual items would be mailed to the users. When using the SQ and ZZ websites, the users also had the option to express their feelings by using “thought bubbles” (SQ) and “whamz” or “shoutz” (ZZ). The SQ and ZZ websites also contained an avatar feature in which the user could customize their look. The MB website tracked activity using “moves,” steps, and miles.

### Behavior Change Techniques

In order to map each device or website onto specific BCTs for promoting PA, we used a previously developed taxonomy for “health and fitness” mobile phone apps [17] that have been recently updated [15]. The taxonomy draws from a number of health behavior theories [18], theory of reasoned action [19] and theory of planned behavior [20], and combines similar theoretical constructs into 16 broad categories with 93 specific BCTs listed within the broader categories. A total of 36 specific BCTs were included for this project, since not all techniques in the full taxonomy applied to children and/or PA promotion. Using our list of 36 BCTs, two researchers assessed the presence of each BCT for each website or device (product) and noted where this BCT was evident. After the independent content analyses, the two researchers conferred and resolved any discrepancies.

### Cognitive Interviews and Survey

To assess the children’s ability to navigate the websites, we used a combination of semi-structured cognitive interviews during website navigation and structured survey responses to gauge the comprehension of website features. The cognitive interviews used a “talk aloud” technique where participants navigated each device website while talking aloud about their understanding of the display, their likes or dislikes about the format, and their understanding of how to navigate the website. The order of which websites were presented to the child during each interview was random. The interviews were video- and audiotaped to provide an accurate account of each participant’s experience. Videotaping was conducted with a digital video camera pointing over the child’s shoulder so that the child’s face was not seen. The audiotape from each session was transcribed.

After the interview, participants also completed a survey to provide feedback about specific website features and their experience using the devices. To ensure comprehension, all questions and response options were read out loud to all participants and researchers marked their responses on the survey. PA was defined for the child as, “any play, game, sport, or exercise that gets you moving and breathing harder,” before answering any questions.

Participants answered questions about their usage (5 questions) and enjoyment of (4 questions) specific website features. When asking about usage of specific features, children chose from the options “never,” “a little,” or “a lot.” For example, one question asking about a SQ specific feature was, “How often did you interact with other people on the Sqord website (high fives, squaks)?” In order to gauge the degree to which a participant enjoyed specific features of the device, they were asked 4 questions for each website; “Tell us how much you liked these features from the Sqord website: (1) Track your PA, (2) Find out about other activities and games, (3) squaks or high fives, and (4) Creating and changing your avatar.” Similar questions were asked for the MB (no avatar) and ZZ website features. Response options included, “disliked,” “okay,” “liked,” or “I don’t know.” We also assessed the participant’s experience as a whole, not related to a specific device, using five additional questions.

### Statistical Analyses

The degree to which each BCT was incorporated into the design of the product was assessed by noting the number of times the BCT category was marked for the device and/or website (assigned 1 point for each subcategory). Also, BCTs that were visible on the home page of the device website or on the tracker itself (eg, display on MB) were assigned an additional point (2 points total) since they would be most visible to the users. The points for each BCT were summed for each product to quantify its overall presence and total scores were compared. Qualitative data analysis was conducted using NVivo 10.0 software (QSR International, Victoria, Australia) to identify common themes mentioned during the cognitive interviews. The transcripts of each cognitive interview were uploaded to NVivo, and one researcher created specific nodes to identify common themes and categories to organize and refine the data. Direct quotes (deidentified) were extracted from the transcriptions to represent general themes. Responses to survey questions were analyzed using descriptive statistics and chi-square analyses (significance was set at *P*<.05). Quantitative data were analyzed using Stata 13.1 (StataCorp LP, College Station, TX, USA).

## Results

### Behavior Change Techniques

**Figure 3:**
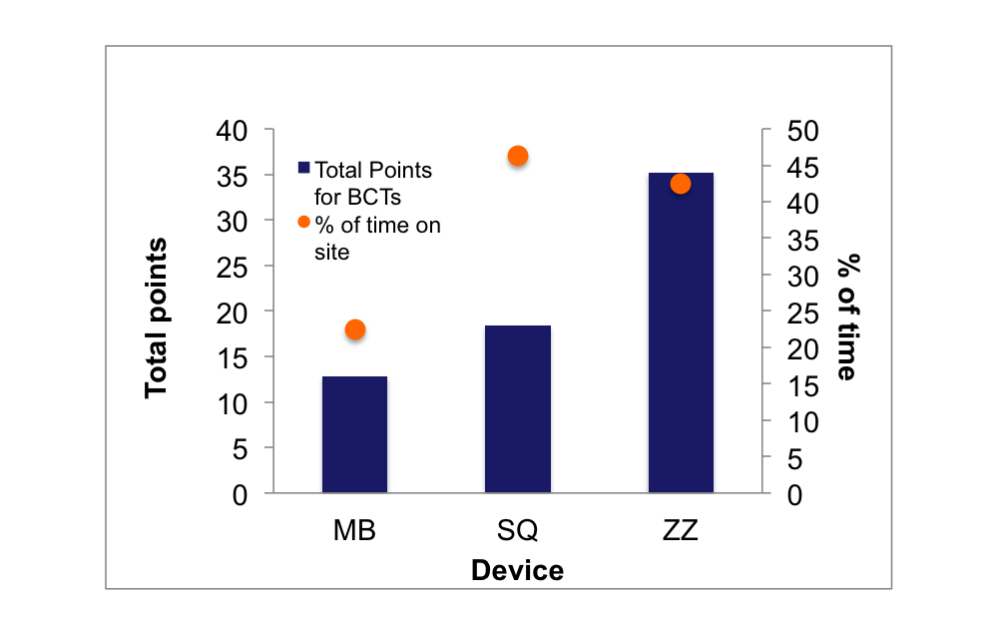
Summary data from behavioral change technique (BCT) scores adjusted for location on the device or website (points) and cognitive interviews (% of time on each site). MB: Movband; SQ: Sqord; ZZ: Zamzee.

Of the possible 36 BCTs, the MB, SQ, and ZZ websites included 8, 15, and 14 techniques, respectively (Multimedia Appendix 1). When total points were adjusted for location on the device and/or website, the MB, SQ, and ZZ scored 16, 23, and 44 points, respectively. Although the participants spent slightly more time on the SQ website during the cognitive interviews, the ZZ website scored the highest total points in regard to presence of BCTs (Figure 3). There were constructs from the taxonomy that were not represented by any of the devices or their associated websites including natural consequences (information about health consequences), repetition and substitution (forming new habits), comparison of outcomes (to a credible source), and antecedents (adding and removing objects to the environment).

### Website Social Support Features

Two websites (SQ and ZZ) contained several social support features, including an avatar that the user could create to represent themselves on the website. No significant differences were observed in the participants’ reactions to the social support features provided by the SQ and ZZ (*P*=.21 to .37; Table 1). Participants reported that they “liked” creating and changing their avatar (SQ: 12/15, 75%; ZZ: 15/16, 94%). Approximately 17% of the cognitive interview time was in reference to the social support features (eg, avatar, clubhouse, high fives, shoutz, squaks), with the avatar being the most popular feature among the participants.

I like how you can “like” things because then it makes me feel good when people like my stuff because it makes me feel happy.

Girl, Age 10, ZZ website

I like the ones (websites) where you get to do a lot of activities.

Boy, Age 9, SQ and ZZ websites

Additionally, the social interactions that took place on the website were an area of interest for some of the participants.

I like finding people. And I like looking at how much points I have and they have.

Boy, Age 6, ZZ website

All of the websites featured BCTs associated with goals and planning, feedback and monitoring, comparison of behavior, and self-belief. Related to these constructs, 17% of the interviews contained speech on self-monitoring techniques including activity charts or points, medals, “moves” or steps or miles, “sqoins,” “zamz,” and minutes in the “zamzone.” All of the websites had a behavioral monitoring feature(s) (eg, charts for tracking activity), but the percentage of participants who indicated “liking” that feature varied by website (MB: 8/16, 50%; SQ: 6/16, 38%; ZZ: 11/16, 69%).

I really like that, um, you get rewards. That’s pretty awesome. I like all the rewards. I like everything, but I think they could make the challenges a bit more clear, like of what you’re supposed to do.

Girl, Age 9, ZZ website

It was apparent that age and maturity modified the participant’s perceptions of the devices or websites as well as their ability to navigate the websites on their own. Most of the participants first navigated to a feature that was located on the home page (MB: 16/16, 100%; SQ: 10/16, 63%; ZZ: 14/16, 88%). Many of the younger children were unfamiliar with using a computer and relied on their parent for guidance. Some aspects of the websites were unclear to a few of the participants and may have hindered the impact of certain BCTs.

I sometimes look at the challenges but I don’t really know what they are.

Boy, Age 7, ZZ website

I don’t really know how to get Sqoins and so it’s really, really confusing how to get sqoins and stuff because, I mean, I did the exercise.

Girl, Age 10, SQ website

And the thing that I didn’t like about this one was that it wasn’t clear and I still don’t know how you get Zamz, which are like the money

Girl, Age 10, ZZ website

On average, 18% of the total interview time was spent on the MB site and its features. The participants spent significantly more time on the SQ and ZZ websites (37% and 34%, respectively). The remaining interview time was spent between websites or additional unrelated commentary. No social support features were observed for the MB and when talking about the MB website, most of the participants mentioned that they did not find the website interesting.

This one I thought was the least interesting because you could barely do anything.

Girl, Age 10, MB website

The MovBand I didn’t really like it because you couldn’t really do anything active, you just look at it which wasn’t really fun to me. It did have one advantage which was seeing if you’re average or not average.

Boy, Age 9, MB website

That was my favorite watch, but least favorite website.

Boy, Age 10, MB website

**Table 1:** Survey responses (percent) related to social support website features for the Sqord and Zamzee. The MovBand contained no social support features and therefore was not included is these analyses.

Social support website feature		Sqord	Zamzee
**Avatar (***P* **=.21)**			
	Disliked	6	0
	Okay	0	0
	Liked	75	94
	I don’t know^a^	19	6
			
**Social interaction (***P* **=.37)**		(squaks, high fives)	(shoutz)
	Disliked	6	0
	Okay	13	25
	Liked	38	38
	I don’t know^a^	44	37
**Frequency of interaction with other users (***P* **=.34)**			
	Never	50	38
	A little	12	37
	A lot	38	25

^a^The response option “I don’t know” was not included in the chi-square analyses.

However, a few participants noted the simplicity of the site made it easy to navigate and understand.

This was my favorite because it was kind of easy to understand. It wasn’t confusing at all.

Boy, Age 9, MB website

When asked if the monitor and website “made me more physically active,” a majority of the participants answered “yes” (MB: 12/16, 75%; SQ: 12/16, 75%; ZZ: 15/16, 94%). When asked about their activity level during the four days of tracker wear, 7 of the 16 participants (44%) reported that they were “more active than usual” and 9 of the 16 participants (56%) claimed that their activity level was “about the same as usual.” Participants also claimed that wearing the monitors during the 4-day study period made them spend more time on the computer (11/16, 69%); therefore, increasing screen time during the study period. Additionally, 69% (11/16) of users said that wearing the monitors made them “enjoy PA more.” Finally, each participant was asked, “If your friends were also wearing these devices, would that make you want to wear them more or less?” and 75% (12/16) responded that they would want to wear them more.

## Discussion

### Principal Findings

The aims of this study were to (1) perform a content analysis to evaluate the BCTs present in three commercially available activity trackers marketed to children and (2) obtain information on the children’s perceptions of these devices and the associated websites using quantitative and qualitative analyses. When the presence of BCTs was summed from each device or website combination, all three devices exhibited less than 50% of the highest possible score (8, 15, and 14 out of a total 36 possible BCTs). Compared with the MB, the SQ and ZZ websites are more child-centric and also utilized more of the BCTs from the taxonomy [11,17]. In addition, children liked the SQ and ZZ websites more than the MB website.

The website layout and design seemed to have an effect on the children’s perceptions of the device. This could possibly be explained by the design of the SQ and ZZ websites being colorful, bright, and “child-friendly.” The MB website was relatively straightforward and monochromatic. Although such a design esthetic may be appealing to adults, it could potentially decrease a child’s interest in using the tracker (Figure 2). It would seem ideal to develop a website that was stimulating enough to encourage consistent use of the tracker while not so engrossing that the child would then spend excess time in front of a screen.

It was apparent that in this study the older children were more comfortable navigating the websites independently, whereas the younger children relied on their parents or guardians for guidance and assistance. Because these devices are marketed as child-friendly, the website design should be appropriate for the user demographics. The SQ and ZZ websites included several links within each page and multiple tabs along the top of the screen that some children did not easily access. A simple and direct navigation platform that targets the most important aspects of the website’s features according to successful BCTs would be beneficial, particularly for children.

The participants enjoyed playing with the avatar feature on the SQ and ZZ sites and spent a considerable portion of their interview talking about this specific feature. However, if the goal of these devices is to increase PA, the primary focus should perhaps be on the activity charts and portions of the website devoted to increasing PA and attempting to also comply with screen time guidelines of less than 2 h/day [21]. One recommendation is to allow user access for an allotted amount of time each day in an effort to support engagement in real-world physical activities [22].

It is recommended that child-centric activity tracker websites be developed in an effort to target BCTs, especially if being used as an intervention tool. Placing essential features on the home page would attract the attention of the users (eg, activity charts). We recognize that incorporating all areas of the taxonomy would pose a challenge to website developers by specifically providing information about why children should engage in PA (natural consequences) and how to make PA part of daily life (repetition and substitution) may be important techniques in promoting behavior change in youth. However, it is not clear that embedding these areas of the taxonomy would actually be appropriate for this age group because of the inability for children to understand such higher level thinking. Natural consequences and repetition or substitution were two of the areas not represented in the content analysis for any of the websites. The recently developed video game “Escape from Diab” [23] incorporated the use of several other theoretical frameworks such as self-determination theory [24] and behavioral inoculation [25] to determine mediators and behavior change processes. Behavioral scientists and website designers worked together to create a video game that would be entertaining and theoretically sound. This type of collaboration should be replicated when attempting to change behavior with the use of technology.

### Limitations

One limitation to this study is the small, relatively active, and nondiverse participants (100% Caucasian). An additional limitation is that we did not collect socioeconomic data for our participants. The participants wore the devices independently from each other, so we do not have data regarding the use of the devices in classrooms or other group settings. The children’s perceptions of the monitors and websites were likely affected by the length of time they spent exploring the features of the devices and their corresponding websites. Children were instructed to spend a minimum of 10 min/day using each website; however, even with parental involvement, we were not able to obtain accurate estimates of website exposure on each of the 4 days despite asking the parents to provide a written log. In addition, our participants wore all three trackers concurrently and this could have affected their use of the trackers and interaction time with the websites. Additional research should test tracker and website usage for each of these devices separately.

It should be noted that all of these devices can be used in a group setting by establishing groups or teams through the website. For this project, we explored our aims from the standpoint of a single user, rather than a team or classroom group. According to the survey results, 75% of the participants indicated that they would want to wear the devices more if their friends were also using them. Therefore, utilizing the “group” features of the websites may encourage the use of these activity trackers more regularly in this population. Since we did not use these features on the devices, we do not know if using these features would have influenced the children’s perceptions of the devices and websites. In addition to not using the group features, the MB website was not seen as visually appealing for the children in this study compared with the others (Figure 2). Future research should explore the use of this feature for each of the devices.

A growing number of studies have examined the use of activity trackers and mobile device apps for stimulating behavior change in adults [6-10,16], but there is a lack of information regarding use of these trackers in younger populations. To our knowledge, this is the first independent research to assess the BCTs in these child-oriented devices in combination with a cognitive interview where we received feedback from the users about their experience with these devices and websites. Although there are far fewer devices specifically targeted to children, the potential exists for manufacturers of adult activity trackers to expand their current offerings to include a child-focused device with a youth version of their existing Web and mobile app content. Therefore, using such devices in school- or group-based interventions would be a logical next step. The use of a criterion measure (such as a research-grade accelerometer) to assess the validity and effectiveness of the activity trackers to increase PA is also recommended. Our results indicate room for expansion in theory-based Web content and changes to website layout so that children are more directly exposed to important and potentially effective BCTs.

### Conclusions

This paper provides the first in-depth content analysis of three child-oriented activity trackers and children’s perceptions of the devices and websites. A growing number of studies have examined the use of activity trackers and mobile device apps for stimulating behavior change in adults, but there is a lack of information regarding use of these trackers in younger populations. Because young children lack the ability to understand abstract concepts or report feelings on surveys, additional techniques were needed to explore the affective responses of young children. The cognitive interviews with a structured qualitative analysis along with the quantitative survey data provide an innovative mixed-methods analysis that deepens our understanding of the children’s affective responses to the devices. With the popularity of the many adult activity trackers and several youth-oriented trackers currently being used in a variety of settings, it is anticipated that manufacturers of adult activity trackers will expand their current offerings to include a child-focused device based on their existing Web and mobile app content. The findings from this study suggest that the trackers or websites that displayed more behavioral constructs were more appealing to the child. However, further research is warranted to explore how the behavior change theoretical constructs are presented on these devices or websites and how these constructs change with long-term use and whether they facilitate healthy behavior change.

## Appendix

Multimedia Appendix 1 Behavior change techniques for activity tracker systems (includes device and website).

## Abbreviations

BCT: behavior change technique
MB: MovBand
PA: physical activity
SQ: Sqord
ZZ: Zamzee
