# Supplementary material for: The Physical Activity Tracker Testing in Youth (P.A.T.T.Y.) Study: Content Analysis and Children’s Perceptions
Source: JMIR Mhealth Uhealth. 2017 Apr 28;5(4):e55. doi: 10.2196/mhealth.6347 (PMC5429435; doi:10.2196/mhealth.6347)
Supplement: Multimedia Appendix 1 [file mhealth_v5i4e55_app1.pdf]

Table 1. Behavior change techniques for activity tracker systems (includes device and website).

BCT[a] category	MOVBand	Sqord	Zamzee
**Goals and planning**			
Goal setting (behavior)	y (charts—home page)	y (sqorcher)	n
Problem solving	n	n	n
Goal setting (outcome)	n	n	n
Action planning	n	y (challenges)	y (challenges)
Review behavior goal(s)	y (charts, pop-up)	y (charts)	y (charts and shoutz)
Discrepancy between current behavior and goal(s)	y (charts)	n	n
Review outcome goal(s)	n	n	n
Commitment	n	y (medals)	y (shoutz, pointz)
**Feedback and monitoring**			
Feedback on behavior	y (digital display on watch)	y (medals)	y (shoutz)
Self-monitoring of behavior	y (chart and watch)	y (charts)	y (chart)
Self-monitor behavior outcome(s)	n	n	n
Biofeedback	n	n	n
Feedback on behavior outcome(s)	n	n	n
**Social support**			
Social support (unspecified)	n	y (club house, challenges, avatar)	y (add friends, avatar)
Social support (practical)	n	n	n
Social support (emotional)	n	y (high fives, squaks)	y (shoutz, whamz)

**Shaping knowledge**				
	Behavior instructions	n	y (1-time tutorial)	n
	Information about antecedents	n	n	n
**Natural consequences**				
	Information—health consequences	n	n	n
	Information—social and environmental consequences	n	n	n
	Monitoring—emotional consequences	n	n	n
	Information—emotional consequences	n	n	n
**Comparison of behavior**				
	Social comparison	n	y (leaderboard—ticker and tab, challenges)	y (leaderboard)
**Associations**				
	Prompts/cues	y (pop-up)	n	n
**Repetition and substitution**				
	Behavior substitution	n	n	n
	Habit formation	n	n	n
	Graded tasks	n	n	n
**Comparison of outcomes**				
	Credible source	n	n	n
**Reward and threat**				
	Nonspecific reward	n	y (avatar)	y (avatar)
	Social reward	n	y (leaderboard—ticker and tab, high fives)	y (leaderboard)
	Reward (behavior)	n	y (medals, points)	y (pointz, zamz)

**Antecedents**				
	Adding objects to the environment	n	n	n
	Removing objects from the environment	n	n	n
**Scheduled consequences**				
	Situation-specific reward	n	y (scorcher, challenges)	y (zamzone, zamz, challenges, rewards)
	Reward incompatible with behavior	n	n	y (eg, X-Box)*
**Self-belief**				
	Focus on past successes	y (dashboard)	y (medals)	y (medals)

[a]BCT: behavioral change technique.
